# Equine dental destructive disorders: an epidemiological survey in northern Germany

**DOI:** 10.3389/fvets.2026.1706621

**Published:** 2026-02-17

**Authors:** M. P. P. Häussling, T. Steinberg, K. Büttner, C. Hannig, M. Hannig, L. Lemke, O. Zierau, C. Staszyk

**Affiliations:** 1 Lüsche Veterinary Clinic, Bakum, Germany; 2 Faculty of Veterinary Medicine, Institute of Veterinary Anatomy, Histology and Embryology, Justus-Liebig-University Giessen, Giessen, Germany; 3 Unit for Biomathematics and Data Processing, Justus- Liebig-University Giessen, Giessen, Germany; 4 Faculty of Medicine Carl Gustav Carus, Policlinic of Operative Dentistry, Periodontology and Pediatric Dentistry, Technische Universität/University of Technology Dresden, Dresden, Germany; 5 Clinic of Operative Dentistry, Periodontology and Preventive Dentistry, University Hospital, Saarland University, Homburg, Germany; 6 Faculty of Biology, Environmental Monitoring & Endocrinology, Technical University Dresden, Dresden, Germany

**Keywords:** periperal caries, infundibular caries, diastema, dental cementum, tooth fracture, apical infection

## Abstract

**Introduction:**

Destructive lesions of the peripheral and infundibular cementum are increasingly recognized in equine dentistry. While similarities to human caries have been noted, current evidence does not conclusively support this classification. This study aims to assess the prevalence, distribution, and potential risk factors associated with these lesions in a defined horse population.

**Materials and methods:**

A total of 114 horses from northern Germany underwent standardized oral examination between 2021 and 2023. Clinical data on peripheral and infundibular lesions, diastemata, and influencing factors such as age and sex were collected and statistically analyzed using adapted grading systems and multiple comparative tests.

**Results:**

Peripheral cemental lesions were present in 77.2% and infundibular changes in 61.4% of the horses. While molars were more frequently affected than premolars, no significant correlation was found between the two lesion types. Infundibular disease increased significantly with age, whereas peripheral lesions showed a non-significant age-related trend. Female horses exhibited a higher prevalence of peripheral cemental lesions.

**Discussion/conclusion:**

Despite similar cemental involvement, peripheral and infundibular lesions differ markedly in clinical relevance and progression, necessitating distinct diagnostic approaches. Peripheral changes are often benign, whereas infundibular lesions may lead to pulpitis or fractures. These findings underline the need for species-specific diagnostic criteria and further research into modifiable risk factors.

## 1 Introduction

The destruction of mineralized dental substances is a significant problem in human medicine and has been recently recognized as an increasing problem in domestic horses (1). Besides mechanical (abrasion and attrition) and chemical (erosion) processes that lead to hard-substance destruction and loss (2), dental caries has been identified as a significant demineralizing dental disease that causes hard-substance destruction and severe endodontic disorders (3). Dental caries is a destructive bacterial disease that potentially affects all teeth of a dentition and all calcified dental tissues, i.e., cementum, enamel, and dentine (4). Although numerous studies have elucidated in detail the underlying pathomechanisms of human caries, it has so far only been described very vaguely whether the hard-substance losses observed in the clinical crown of equine teeth actually correspond to human caries. Nevertheless, destructive dental diseases in horses are uncritically referred to as “equine caries” in the relevant literature [e.g., see Borkent et al. (5), Jackson et al. (4), and Gere and Dixon (3)]. In view of the uncertain scientific data, we prefer to use the terms ‘destructive disorder/disease’ or “caries-like disease” instead of “caries” in the following text to describe related pathological changes in the equine dentition.

Considering the striking difference between brachydont teeth of humans and hypsodont teeth of horses, the transfer of conclusions in human caries research to the equine species is not possible without critical consideration of the equine specific dental morphology. Hitherto, equine dental destructive disorders have been reported exclusively for cheek teeth (6), and thus the following appraisal of equine-specific dental features is restricted to the equine maxillary and mandibular cheek teeth. In humans, the dental crown is completely covered by enamel and throughout life exposed in the oral cavity. In contrast, equine teeth experience a continued occlusal wear compensated by a lifelong dental eruption. The portion of the tooth that is visible in the oral cavity is referred to as the clinical crown—topographically synonymous with the crown of the brachydont tooth (7). However, from a morphological perspective, the clinical crown of equine teeth features a completely different anatomy compared to the crown of brachydont teeth. The occlusal surface shows a complex layered histologic structure composed of enamel ridges, dentinal basins, and cemental areas. The peripheral surfaces of the equine clinical crown are completely covered by dental cementum. A noteworthy equine dental characteristic is the presence of two different types of dental cementum exposed to the oral environment. The equine peripheral cementum shares structural similarities with the cementum covering the roots of brachydont teeth. Nevertheless, the peripheral cementum on the clinical crowns of cheek teeth in horses shows a thickness of several millimeters instead of less than 100 μm reported for human dental roots. A unique type of dental cementum is present in the infundibula of the equine dentition (8). Each maxillary cheek tooth is equipped with a mesial and a distal infundibulum. Infundibula are best described as enamel invaginations that extend from occlusal in an apical direction. During dental development underneath the oral mucosa, the dental follicle reaches into the infundibula and initiates dental cementum formation. However, the occlusal entrance of the infundibulum becomes exposed after dental eruption and consequently the occlusal vascular supply is interrupted. This developmental condition explains the porous microscopic structure of the equine infundibular cementum featuring an ample network of empty blood vascular channels and cementoblast lacunae (9). Unsurprisingly, the peripheral as well as the infundibular cementum are described as the most susceptible dental substances of the equine clinical crown to the onset of destructive disorders, uncritically referred to as peripheral and infundibular “caries.” Accordingly, it is imperative to focus the scientific analysis of destructive crown diseases on the different substances in humans (enamel) and horses (peripheral cementum and infundibular cementum).

Previous studies dealing with equine dental destructive disorders were mainly focused on epidemiological surveys and obtained very heterogenous results that indicated a wide range of prevalence for peripheral “caries.” The lowest prevalences (0.9 to 6.1%) were reported in Sweden (3), while the highest prevalences (91%) were documented in Scotland (10). In the United Kingdom [51%, (5)] and Western Australia [59%, (11)], intermediate prevalence rates were reported. A study on infundibular caries reported a prevalence of 45.5% in the United Kingdom (12). Similar studies focused on the prevalence of infundibular caries in donkeys. These studies reported prevalence rates of 8–100% in Inner Mongolia (depending on the region) and 27.8% in the United Kingdom (13).

Our aim was to determine the prevalence of destructive disease of the peripheral and infundibular cementum in a locally defined horse population in Germany. To identify possible predisposing factors and create a basis for further histopathological investigations, supplemental data were collected, i.e., topographical data concerning the position of lesions (within the dentition and within a tooth), diagnoses of additional dental disorders, and possible influential factors such as age and sex.

## 2 Materials and methods

### 2.1 Participants

A total of 114 horses were investigated from September 2021 to March 2023 (Table 1). Most animals were referred to a veterinary clinic located in northern Germany (Tierklinik Lüsche GmbH), while only a minority were examined at their respective home stable. The primary reason for veterinary visit was routine dental assessment, although a subset of patients required intervention for dental conditions such as extractions. Other clinical concerns included unilateral nasal discharge, headshaking, and chewing abnormalities. To maintain geographical consistency, only equines residing in northern Germany for at least 1 year and within a radius of 200 km of the clinic were considered eligible for inclusion in the study. The average age of the surveyed horses was 11.1 years, ranging from 3 to 26 years. The horses were categorized into four age groups: 3–5 years old (*n* = 27, 23.7%); 6–9 years old (*n* = 26, 22.8%); 10–15 years (*n* = 29, 25.4%); and >15 years old (*n* = 32, 28.1%).

**Table 1:** Patient data (age, breed, sex) and pathological findings, i.e., lesions in the peripheral cementum (LPC), lesions in the infundibula (LI) and occurrence of diastemata within the cheek tooth rows.

Horse number	Age (years)	Breed	Sex	LPC	LI	Diastemata
1	3	Warmblood	Stallion	Yes	No	No
2	3	Warmblood	Stallion	Yes	No	No
3	3	Warmblood	Stallion	No	No	No
4	3	Warmblood	Stallion	No	No	No
5	3	Warmblood	Stallion	No	No	No
6	3	Warmblood	Stallion	No	No	No
7	3	Warmblood	Stallion	No	No	No
8	3	Warmblood	Stallion	No	No	No
9	3	Warmblood	Stallion	No	No	No
10	3	Warmblood	Stallion	No	No	No
11	13	Warmblood	Gelding	Yes	No	No
12	5	Warmblood	Gelding	Yes	No	Yes
13	13	Warmblood	Gelding	Yes	No	Yes
14	11	Warmblood	Mare	Yes	No	No
15	15	Warmblood	Gelding	No	Yes	Yes
16	4	Warmblood	Mare	Yes	No	Yes
17	18	Friesian horse	Mare	Yes	No	No
18	17	Friesian horse	Mare	Yes	No	Yes
19	8	Quarter horse	Gelding	Yes	No	No
20	5	Warmblood	Mare	Yes	No	No
21	11	Warmblood	Gelding	No	No	Yes
22	8	Warmblood	Gelding	No	No	Yes
23	11	Warmblood	Gelding	Yes	Yes	No
24	14	Warmblood	Gelding	Yes	Yes	Yes
25	8	Warmblood	Gelding	No	No	Yes
26	4	Warmblood	Gelding	Yes	No	Yes
27	15	Warmblood	gelding	Yes	Yes	Yes
28	10	Warmblood	Mare	Yes	Yes	Yes
29	7	Quarter horse	Gelding	Yes	Yes	Yes
30	10	Pony	Gelding	No	No	No
31	21	Warmblood	Mare	Yes	Yes	Yes
32	15	Warmblood	Gelding	Yes	Yes	Yes
33	10	Warmblood	Gelding	Yes	Yes	Yes
34	17	Warmblood	Gelding	Yes	No	Yes
35	14	Warmblood	Mare	Yes	Yes	Yes
36	19	Warmblood	Gelding	Yes	Yes	Yes
37	6	Warmblood	Gelding	Yes	Yes	Yes
38	7	Warmblood	Gelding	Yes	Yes	Yes
39	9	Quarter horse	Mare	Yes	Yes	No
40	15	Warmblood	Mare	Yes	Yes	Yes
41	8	Warmblood	Mare	Yes	Yes	Yes
42	8	Friesian horse	Gelding	Yes	No	No
43	12	Warmblood	Stallion	Yes	Yes	Yes
44	4	Warmblood	Mare	Yes	No	Yes
45	9	Warmblood	Gelding	No	No	No
46	6	Warmblood	Gelding	Yes	No	No
47	19	Warmblood	Stallion	No	Yes	No
48	6	Warmblood	Gelding	Yes	No	No
49	7	Warmblood	Stallion	No	Yes	No
50	3	Warmblood	Gelding	Yes	No	No
51	14	Warmblood	Gelding	Yes	Yes	Yes
52	6	Warmblood	Mare	No	No	No
53	7	Warmblood	Gelding	No	No	No
54	6	Warmblood	Gelding	No	Yes	No
55	5	Warmblood	Mare	No	Yes	No
56	13	Warmblood	Gelding	Yes	Yes	Yes
57	7	Warmblood	Gelding	Yes	Yes	No
58	3	Warmblood	Mare	Yes	No	Yes
59	9	Warmblood	Gelding	Yes	Yes	Yes
60	3	Warmblood	Stallion	Yes	No	No
61	6	Warmblood	Mare	Yes	No	Yes
62	19	Warmblood	Mare	Yes	No	Yes
63	3	Warmblood	Mare	Yes	No	Yes
64	10	Warmblood	Mare	No	No	No
65	17	Friesian horse	Gelding	Yes	Yes	No
66	6	Warmblood	Mare	No	Yes	No
67	8	Spanish horse	Gelding	Yes	No	Yes
68	15	Warmblood	Gelding	Yes	No	No
69	9	Friesian horse	Stallion	Yes	Yes	Yes
70	7	Warmblood	Mare	Yes	Yes	Yes
71	8	Friesian horse	Mare	Yes	Yes	Yes
72	5	Paint horse	Gelding	Yes	Yes	No
73	9	Warmblood	Mare	Yes	Yes	No
74	3	Warmblood	Mare	Yes	No	No
75	10	Warmblood	Gelding	Yes	Yes	No
76	13	Pony	Gelding	Yes	Yes	No
77	5	Warmblood	Mare	Yes	Yes	No
78	11	Warmblood	Mare	Yes	Yes	Yes
79	10	Warmblood	Gelding	Yes	Yes	Yes
80	13	Pony	Gelding	Yes	Yes	No
81	24	Pony	Gelding	No	Yes	Yes
82	12	Warmblood	Mare	Yes	Yes	No
83	3	Warmblood	Gelding	Yes	No	No
84	4	Warmblood	Mare	Yes	Yes	Yes
85	10	Spanish horse	Gelding	Yes	Yes	Yes
86	4	Warmblood	Mare	Yes	Yes	Yes
87	14	Friesian horse	Gelding	Yes	Yes	Yes
88	13	Warmblood	Mare	Yes	Yes	Yes
89	5	Warmblood	Gelding	No	Yes	Yes
90	6	Warmblood	Mare	Yes	Yes	Yes
91	19	Warmblood	Mare	Yes	Yes	Yes
92	19	Pony	Mare	Yes	Yes	Yes
93	18	Warmblood	Gelding	Yes	Yes	Yes
94	17	Warmblood	Mare	Yes	Yes	Yes
95	23	Quarter horse	Mare	Yes	Yes	Yes
96	24	Pony	Mare	Yes	Yes	Yes
97	22	Warmblood	Gelding	Yes	Yes	No
98	15	Warmblood	Gelding	Yes	Yes	No
99	18	Warmblood	Gelding	Yes	Yes	No
100	18	Warmblood	Gelding	Yes	Yes	No
101	22	Warmblood	Gelding	No	Yes	No
102	21	Pony	Gelding	Yes	Yes	Yes
103	24	Pony	Mare	Yes	Yes	Yes
104	17	Paint horse	Mare	Yes	Yes	Yes
105	26	Pony	Gelding	Yes	Yes	Yes
106	18	Warmblood	Mare	No	No	Yes
107	18	Warmblood	Mare	Yes	Yes	Yes
108	16	Warmblood	Mare	Yes	Yes	Yes
109	16	Warmblood	Mare	Yes	Yes	Yes
110	15	Warmblood	Gelding	Yes	Yes	Yes
111	22	Warmblood	Gelding	Yes	Yes	Yes
112	15	Warmblood	Gelding	Yes	Yes	Yes
113	18	Warmblood	Gelding	Yes	Yes	No
114	16	Pony	Mare	Yes	Yes	No

### 2.2 Data collection

During the examination, the owners were asked to provide consent for the collection and publication of data related to their horses. A custom-designed questionnaire tailored for this survey was employed, encompassing inquiries regarding the horse’s age, place of residence, sex, breed, utilization, and dental history/ailments. In instances where the owner was not physically present at the clinic, the questionnaire was administered via telephone interview.

Destructive disease of the peripheral cementum and infundibular cementum was documented using a specially crafted examination form for oral evaluations. Destructive disease of the peripheral cementum was noted on both sides (lingual/palatal and buccal) of all cheek teeth, and the destructive disease of the infundibular cementum was documented in all maxillary cheek teeth. Destructive changes were categorized based on severity according to a grading system as described below. The presence of diastemata was documented as soon as food impaction was recognizable in the interdental space. Each diastema was recorded according to the specific interdental space, with the location identified using the Triadan position of the two adjacent teeth. No additional categorization based on diastema size and/or shape was performed. Other pathological changes, such as tooth loosening and loss, dental crown fractures, and malocclusions such as wave mouth and dental hooks, were systematically documented. For oral examination, horses were sedated while standing, employing a combination of butorphanol (0.01 mg/kg) and detomidine (0.01 mg/kg). A full-mouth speculum was inserted to facilitate the examination with a water lavage to remove food debris. The visual detection and categorization of caries-like lesions was carried out by using an oral endoscope, a headlight, and a dental mirror. Subsequently, the investigation was supplemented and completed by palpation. Simultaneously, a second person recorded all relevant information, such as the location of findings on the teeth and the presence of other pathological conditions.

### 2.3 Classification of destructive lesions of the peripheral cementum

**Figure 1:**
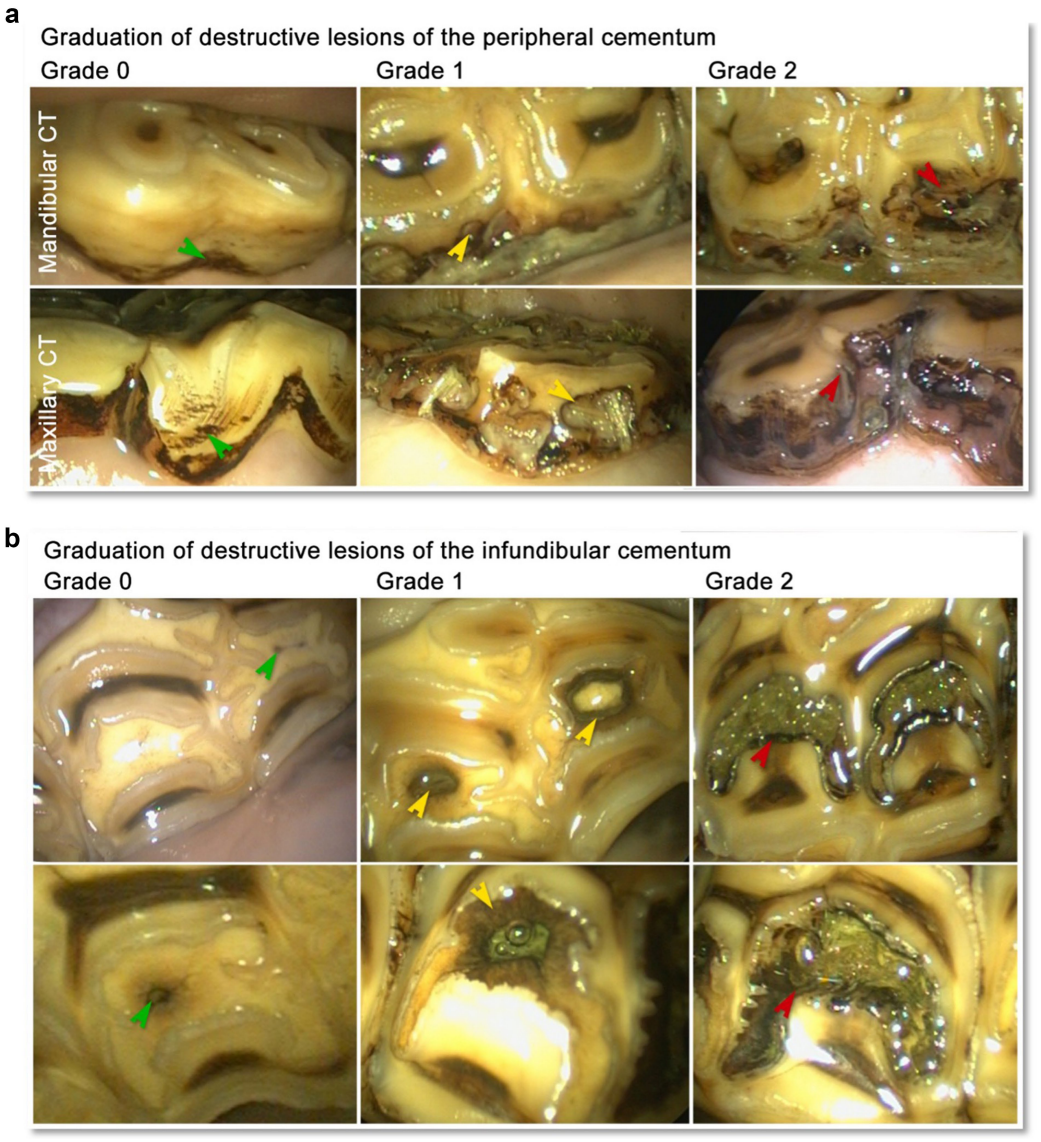
**(a)** Lesions of the peripheral cementum in equine cheek teeth (CT). Grad 0: Peripheral cementum featuring resorptive lacunae and brownish discoloration (green arrow heads). Grad 1: Peripheral cementum featuring discolorate resorptive lacunae extending into the peripheral enamel (yellow arrow heads). Grad 2: Peripheral cementum showing discolorate resorptive lacunae extending through the peripheral enamel into the dentin (red arrow heads). **(b)** Lesions of the infundibular cementum in equine maxillary cheek teeth. Grad 0: Infundibular cementum featuring vascular canals < 2 mm (green arrow heads) and mild discoloration in the surrounding of the vascular canal. Grad 1: Infundibula featuring a vascular canal >2 mm and/or discoloration of the infundibular cementum up to the infundibular enamel (yellow arrow heads). Grad 2: Infundibula featuring discoloration of infundibular cementum and of the infundibular enamel (red arrow heads) as well as focal discoloration of the periinfundibular dentin.

A grading system according to Honma’s grading system as modified by Dacre (14) was modified and used to grade destructive lesions of the peripheral cementum (Table 2 and Figure 1).

**Table 2:** Grading system for the classification of destructive disease of the peripheral cementum in equine cheek teeth.

Grade	Definition
0	Teeth without structural abnormalities of the peripheral cementum, with or without discoloration (yellow/brown/dark brown)
1	Teeth with palpable, discolorate resorptive lacunae in the peripheral cementum or additional extending into the peripheral enamel
2	Teeth with palpable, discolorate resorptive lacunae extending into the dentin

### 2.4 Classification of destructive lesions of the infundibulum

A grading system according to Honma’s grading system as modified by Dacre (14), was modified to grade destructive lesions of the infundibulum (Table 3 and Figure 1a).

**Table 3:** Grading system for the classification of destructive disease of the infundibulum in equine maxillary cheek teeth.

Grade	Definition
0	Infundibula without textual or color changes, vascular canal < 2 mm
1	Infundibula with color changes and/or widening of the vascular canal (>2 mm), changes restricted to infundibular cementum and infundibular enamel
2	Infundibular lesions extending into the infundibular dentin, vascular canal not recognizable

## 3 Data analysis

Data were organized in an Excel spreadsheet and analyzed using SAS^®^ 9.4 (2nd edition, Statistical Analysis System Institute Inc., Cary, NC, USA). The prevalence rates of destructive dental disease of the peripheral cementum and infundibular cementum were calculated. Teeth showing evidence of one or both forms of disease were classified as affected and included in the destructive disease group. The study compared the frequency of destructive disease of the peripheral cementum between the 12 rostral cheek teeth (Triadan 06–08) and the 12 caudal cheek teeth (Triadan 09–11), as well as between the mandibular and maxillary arcades, using McNemar’s tests. To assess differences in prevalence between the buccal and palatal/lingual sides, a Wilcoxon signed-rank test was applied. Additionally, McNemar’s tests were used to identify the most commonly affected infundibula (106 m, 206 m, 109 m, 209 m, 109d, 209d, 110 m, and 210 m). A Bowker test was conducted to compare the prevalence of two specific infundibula. Each tooth was evaluated individually in these analyses.

To compare diastemata frequency between the maxilla (109/10, 110/11, 209/10, and 210/11) and the mandibula (306/08 and 406/07), McNemar’s test was carried out. To explore potential correlations, a Chi-square test for equal proportions was conducted to examine the relationship between the prevalence of diastemata and the prevalence of the destructive disease of the peripheral cementum. Additionally, the Chi-square test was applied to assess the influence of sex and age as factors.

To show the relationship between destructive disease of the peripheral cementum and diastemata, a separate Excel spreadsheet was created. All diastemas were listed, along with the teeth forming the diastema. The degrees (0, 1, 2) of the four lateral surfaces of the teeth forming the diastema were combined to form a number. The highest degree was the deciding factor. A Chi-square test for equal proportions was used to calculate the correlation between these two diseases.

An additional Excel spreadsheet was created to examine the relationship between destructive disease of the peripheral cementum and of the infundibulum in more detail. All affected infundibula were listed, and it was noted if the corresponding tooth was also affected by destructive disease of the peripheral cementum. The highest degree of both lateral surfaces was selected to obtain a value, and a Chi-square test for equal proportions was used for comparisons.

## 4 Results

### 4.1 Study population

The predominant horse breed was warmblood, making up 78% of the examined horses. Other breeds, such as Friesian horses, various types of ponies, quarter horses, paint horses and Spanish horses, made up 22% (Table 1). Male horses constituted the majority (*n* = 71, 62.3%), including stallions (*n* = 15, 13.2%). The primary reason for a visit to the veterinarian was the routine dental control (62%), followed by reasons unrelated to dental issues (23%) and tooth extractions (15%). Almost all horses featured a complete dentition (93%), while one or more teeth were missing in 7% of the cases.

### 4.2 Prevalence of dental destructive diseases

**Figure 2:**
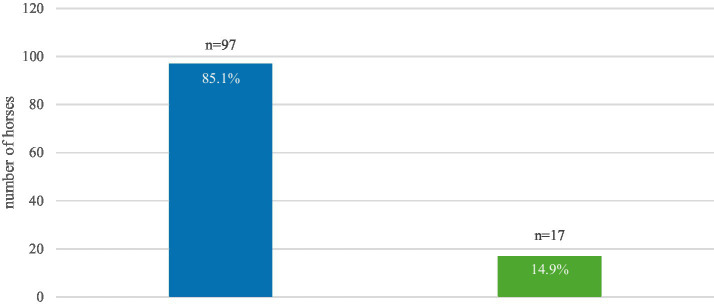
Prevalence of dental destructive diseases in 114 horses. Blue: affected horses, green: horses showing no destructive lesions.

Horses with destructive lesions (85.1%) outnumbered horses without lesions (14.9%) (Figure 2).

### 4.3 Destructive disease of the peripheral cementum

**Figure 3:**
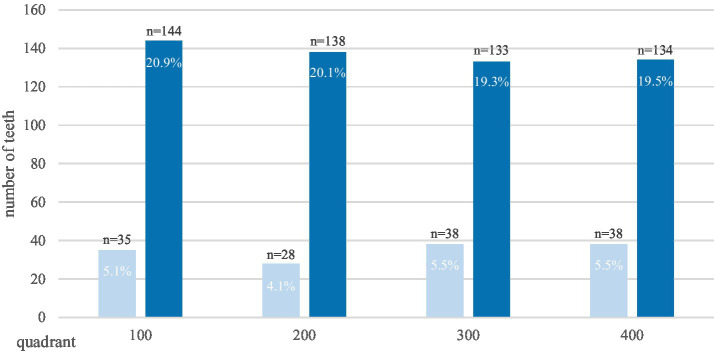
Prevalence of destructive disease of the peripheral cementum in premolar teeth (light blue) and molar teeth (dark blue) within the individual jaw quadrants of 88 affected horses. The absolute numbers and percentages refer to the affected teeth, in total 688.

Among the horses examined, 77.2% (*n* = 88/114) suffered from destructive disease of the peripheral cementum. Molars (79.8%) were about four times more affected than premolars (20.2%) (Figure 3).

For the statistical analysis, a summarization of all quadrants was conducted, with subsequent division into premolars and molars. Grades 1 and 2 were amalgamated into a single value, which was then calculated against the number of teeth exhibiting grade 0. A statistically significant discrepancy in the severity of the conditions was detected (*p* < 0.0039). In 66.2% of cases where premolars were assigned a grade 0, molars were assigned grades 1 or 2, while only 5.4% of cases where molars were assigned grades 1 or 2 were assigned a grade 0 to premolars. This finding suggests that molars are more susceptible to changes than premolars. A non-significant difference in severity was observed between the upper and lower jaw (*p* = 0.0931). The finding indicates the presence of symmetry between the left and right sides of the dentition as well as between the lower and upper arcades.

**Figure 4:**
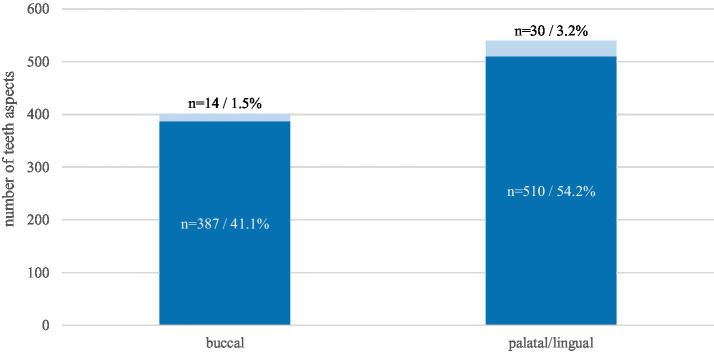
Prevalence of destructive disease of the peripheral cementum on the buccal and palatal/lingual aspect of 88 affected horses. The absolute numbers and percentages refer to the total amount of the aspects of 688 affected cheek teeth. Dark blue: grade 1, light blue: grade 2.

The palatal/lingual aspect of the cheek teeth was more frequently affected (Grade 1: 54.2%, Grade 2: 3.2%) by destructive disease of the peripheral cementum than the buccal aspect (Grade 1: 41.1%, Grade 2: 1.5%) (Figure 4).

For the purposes of the statistical analysis, grades 1 and 2 were summarized. This approach facilitated the calculation of the number of affected teeth, as determined by the total sum per side. A statistically significant difference was identified (*p* < 0.0001) between the buccal and palatal/lingual aspects of the cheek teeth. The median number of affected teeth per horse was determined to be three on the palatal/lingual side, while on the buccal side, the median was two.

### 4.4 Destructive disease of the infundibulum

**Figure 5:**
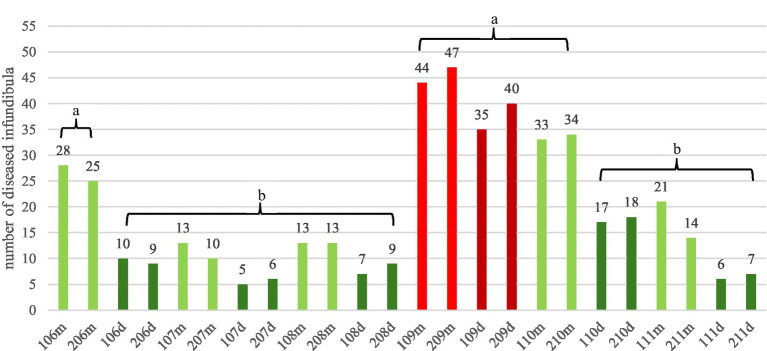
Frequency of destructive infundibular disease (grade 1 and grade 2) of 70 affected horses. The mesial (m) infundibulum is displayed in light colors, the distal (d) infundibulum is displayed in dark colors. The most frequently affected infundibula (tooth 09) are emphasized by red colors. Infundibula labeled with “a” are significantly (*p* = 0.0169) more frequently affected (61.6%) compared to infundibula labeled with “b” (38.4%).

Destructive lesions of the infundibulum were identified in 61.4%, (*n* = 70/114) of the horses. Triadan position 09 exhibited the highest prevalence of 35.8% (*n* = 166 of 464 dieseased infundibula). The second highest prevalence was detected in the Triadan position 10 with 22%, followed by Triadan position 06 (15.5%) (Figure 5). No difference in the prevalence of disease was found between the right and left side of the dentition (*p* > 0.07), indicating symmetry between the two sides.

**Figure 6:**
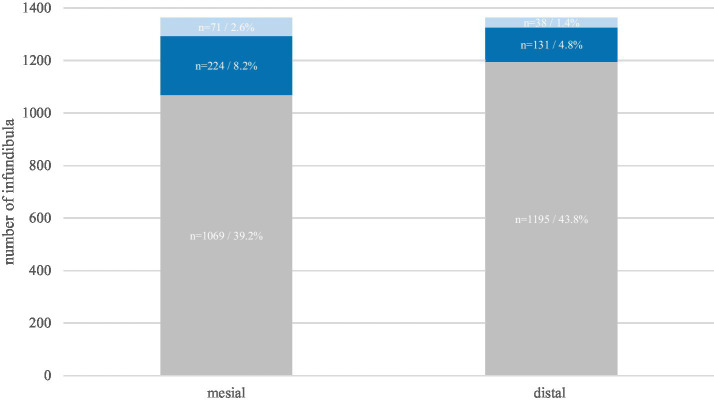
Prevalence of destructive infundibular disease in the mesial and distal infundibulum of 1,364 investigated maxillary equine cheek of 114 horses. The absolute numbers and percentages refer to the number of investigated infundibula (in total 2,728). Gray: grade 0, dark blue: grade 1, light blue: grade 2.

The mesial infundibulum was statistically significantly (*p* < 0.0001) more frequently affected (63.6%) than the distal infunidbulum (36.4%). In both infundibula, grade 1 lesions occurred about three times more frequently than grade 2 lesions (Figure 6).

### 4.5 Diastemata

**Figure 7:**
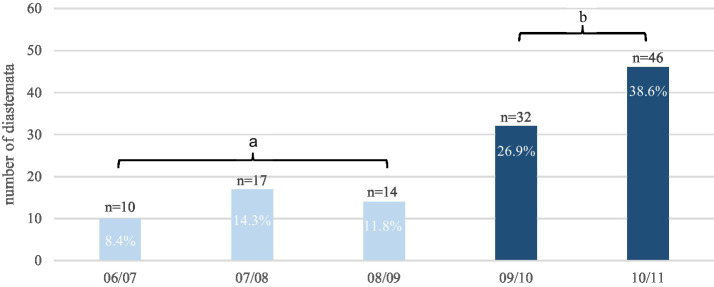
The total number of diastemata (119) observed in the respective interdental spaces of the maxillary arcades of 63 affected horses. Interdental positions labeled with “b” are almost two times more affected (65.5%) compared to interdental positions labeled with “a” (34.5%).

**Figure 8:**
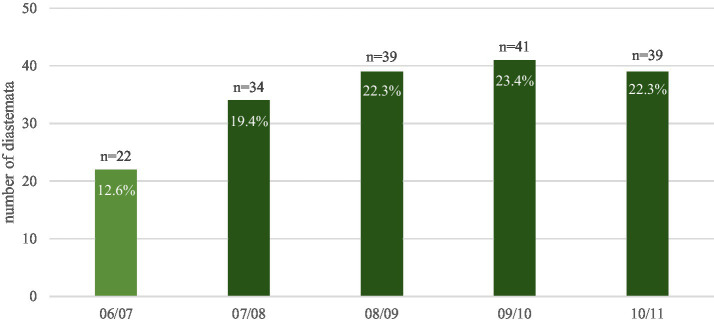
The total number of diastemata (175) observed in the respective interdental spaces of the mandibular arcades of 63 affected horses. The interdental position between the second and third premolar teeth (06/07) is less frequently affected than any of the other interdental positions.

Diastemata were observed in the cheek teeth arcades in 55.7% (*n* = 63/114) of the horses. Mandibular cheek teeth arcades (48.3%) were statistically significantly (*p* = 0.0031) more frequently affected than maxillary arcades (33.3%); 67.1% had no diastemata in the maxilla but had diastemata in the mandible, whereas only 21.1% diastemata in the maxilla but no diastemata in the mandible. The following diagrams illustrate the descriptive statistics for the number of diastemata in the maxillary (Figure 7) and mandibular arcades (Figure 8).

For the purpose of statistical analysis, the number of diastemata in the interdental positions of teeth 09/10 and 10/11 were pooled and compared with the number of remaining diastemata in the maxillary arcades. The results indicated that the selected diastemata were affected significantly (*p* = 0.0015) more often than the others.

In the mandibular arcades, the prevalence of diastemata was distributed in a homogeneous manner from tooth 07 to tooth 11. The interdental position between teeth 06 and 07 was affected significantly (*p* < 0.0001) less often. The statistical calculation is analogous to that of the diastemata in the maxilla. There was no signficant difference in the prevalence of disease between the right and left side right and left side of the head (*p* > 0.1). This means that there is symmetry between the two sides.

### 4.6 Summarized consideration of further findings

Although 58.8% of the horses (*n* = 67/114) exhibited concurrent destructive disease of the peripheral and infundibular cementum, no statistically significant correlation between these pathologies was identified. Furthermore, both diseases were observed on the same tooth in only 28.9% of the affected teeth.

Among all horses, 55.7% (*n* = 63/114), exhibited diastemata and lesions of the peripheral cementum. Among the paired teeth with a diastema, 53.9% exhibited peripheral changes on at least one of the two teeth. However, no statistically significant correlation was identified between the presence of destruction of the peripheral cementum and the presence of diastemata.

### 4.7 Influence of sex and age

Lesions of the peripheral cementum were significantly (*p* = 0.0296) more frequent in female horses (88.4%) than in male horses (70.8%). No significant correlation with sex was detected for the prevalence of destructive disease of the infundibular cementum.

### 4.8 Influencing factor age

**Figure 9:**
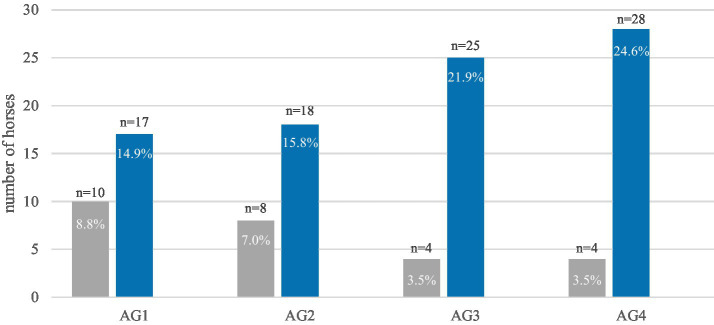
The prevalence of destructive diseases of the peripheral cementum in equine cheek teeth of 114 horses, within the four age groups (AG). The diagram illustrates a notable increase in changes with age. Gray: horses without destructive lesions of the peripheral cementum; blue: horses with destructive lesions (grade 1 and grade 2).

**Figure 10:**
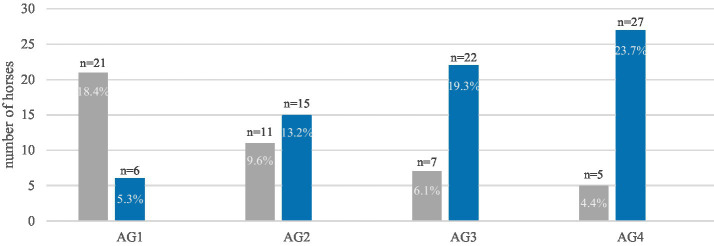
The prevalence of the destructive infundibular disease in equine cheek teeth of 114 horses, within the four age groups (AG). The diagram shows a significant (*p* < 0.0001) increase with age. Gray: horses without destructive lesions of the infundibula, blue: horses with destructive lesions of the infundibula (grade 1 and grade 2).

**Figure 11:**
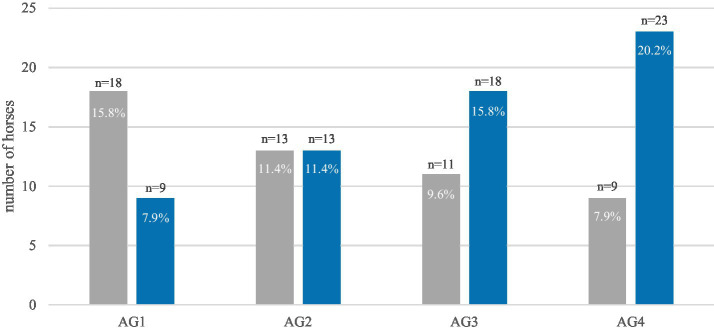
The prevalence of diastemata in equine cheek tooth arcades of 114 horses, within the four age groups (AG). The diagram shows a significant (*p* = 0.0217) increase with age. Gray: horses without diastemata, blue: horses with diastemata.

The prevalence of destructive disease of the peripheral cementum showed an age-related trend (Figure 9). Nevertheless, the relationship was not statistically significant. In contrast, the prevalence of destructive diseases of the infundibulum cementum increased significantly (*p* < 0.0001) with age (Figure 10). Likewise, the prevalence of diastemata increased significantly (*p* = 0.0217) with age (Figure 11).

## 5 Discussion

### 5.1 Epidemiological considerations

Destructive diseases of the peripheral cementum and of the infundibulum in equine cheek teeth have been documented in several countries, such as Australia, Scotland, the United Kingdom, and Sweden. Surprisingly, prevalence rates of destructive disease of the peripheral cementum varied widely, ranging from 6.1% in Sweden (3) to 91% in Scotland (10). Given the very heterogeneous rates among geographic regions, location-dependent factors may be operating. Therefore, it is meaningful to conduct additional local surveys to elucidate general and locally specific factors affecting the disease. Our study supplements data from a region in northern Germany and indicates a prevalence of 77.2% of horses with destructive disease of the peripheral cementum. This rate is more similar to the rates in the United Kingdom [51.7%, (5)], Australia [58.8%, (11)], and Scotland [91%, (10)] than to the reported in Sweden [6.1%, (3)]. Although the comparability of the individual study results is limited due to different methodological approaches (intra vitam vs. postmortem examination, different scoring systems, etc.) and study population sizes (101 to more than 500), it can nevertheless be stated that destructive disease of the peripheral cementum represent a relevant problem with prevalence rates sometimes well above 50%. The situation is similar with destructive disease of the infundibulum with documented rates of 45.5% in the United Kingdom (12) and northern Germany (61.4%). In Sweden (3), the prevalence of destructive disease of the infundibulum is 17%, while the prevalence of destructive disease of the peripheral cementum is significantly lower.

### 5.2 Influential factors—food and saliva

The consumption of a variety of foodstuffs has been linked to an elevated risk of developing destructive dental diseases, usually peripheral and infundibular caries (1, 2, 6, 11, 15, 16), in horses. Critical foodstuffs are concentrates such as cereal, oats, oaten hay and hay/haylage. These foods are characterized by a high proportion of fermentable carbohydrates that are soluble in water, which favors an oral bacterial flora producing high amounts of acidic metabolites. The resulting acidic peridental and intrainfundibular environment causes demineralization of the dental hard tissue, which is clinically diagnosed as a destructive disease of the peripheral cementum and infundibulum (16). Horses fed oaten hay were three times more susceptible to suffering from destructive disease of the peripheral cementum than those not fed oaten hay (6). Furthermore, horses kept on pasture for a minimum of 8 months per year had a reduced prevalence of lesions of the peripheral cementum (6). This finding was explained by two key factors: (1) the lower concentration of water-soluble carbohydrates in the food consumed; and (2) a continuous and sufficient production of acid-buffering saliva throughout pasture feeding (6). In an experimental approach, Jackson et al. (16) changed the diet of 42 horses from oaten hay to hay with a lower water-soluble carbohydrate content and documented a significant reduction of destructive lesions of the peripheral cementum. They suggested that both the feed itself and the duration of feed intake, and thus the quantity of saliva production, are relevant factors influencing the development of equine destructive dental diseases, which is similar to human caries (16, 17). Causative factors known from human caries research should be investigated in horses, including the influence of different types of sugar, the adhesion of the ingested feed, saliva quantity and quality (17).

In addition to the type of feed and the resulting content of water-soluble carbohydrates, the capacity of saliva to buffer acidic pH must be considered as a relevant factor for the onset of destructive dental diseases. In this respect, it is not only the saliva itself that is interesting, but also the question of whether and to what extent saliva varies intra- and interindividually. Lundström et al. (18) documented an increase in the concentration of saliva electrolytes (sodium, potassium, calcium, phosphate and urea) during the chewing process, with the exception of bicarbonate, which is the most crucial component for saliva buffering capacity. However prolonged feeding leads to an increased overall amount of saliva production, resulting in a constantly increasing amount of bicarbonate that facilitates pH neutralization (18). The first indication that saliva has an enormous influence on the horse’s dental health comes from a case report of two horses that suffered from an unilateral injury of the parotid salivary duct (19). Both horses showed a remarkable asymmetry in the degree of peripheral cementum destruction between the affected side (severe peripheral cementum destruction) and the unaffected side. This implies that inadequate saliva production increases susceptibility to the destructive disease of the peripheral cementum and leads to a more severe manifestation of the disease (19). These findings suggest that proximity to the opening of the parotid duct plays a significant role in the development of caries such as lesions, although, at the same time, it can be assumed that the saliva becomes dispersed in the entire cavity by the mastication. These considerations provide a fact-based foundation for an explanatory hypothesis for the significant asymmetry in the prevalence of destructive disease of peripheral cementum, i.e., higher prevalence in molars compared to premolars and on labial/palatal sides compared to the buccal sides. This asymmetry might be explained by the position of the openings of the salivary glands. The duct of the large parotoid gland reaches the oral cavity at the buccal side of maxillary Triadan position 08 (20). The smaller sublingual gland features several openings directed to the lingual side of the mandibular premolars (21). This topography of the salivary openings, together with the typical head position toward the ground during feeding, suggests a higher accumulation of saliva on the buccal sides of the premolars and less accumulation of saliva in other regions of the oral cavity.

### 5.3 Sex

Surprisingly, our study revealed a significantly higher prevalence of destructive disease of the peripheral cementum in the mares (88.4%) than in male horses (70.8%). This finding is in line with the results from other studies [Lee et al. (10), Jackson et al. (11), and Lundström et al. (18)] which also reported a higher prevalence of destructive disease of the peripheral cementum in female horses. Similar sex-related results were obtained in studies on human caries. It has been shown that the oral environment of women is significantly more cariogenic than that of men due to hormone-induced changes of the biochemical composition of saliva and the overall saliva flow (22). Although studies on sex-specific differences in the composition of saliva in horses are lacking, it is quite likely that equine saliva composition is also influenced by hormonal factors in a way that predisposes female horses to the onset of destructive diseases of the peripheral cementum.

### 5.4 Destructive disease of the peripheral cementum vs. destructive disease of the infundibular cementum

The significant increase in destructive infundibular disease with age corroborates the results from a study conducted in the United Kingdom (1). The prevalence of destructive diseases of the peripheral cementum also increases with age, although it was not statistically significant. An explanation for these different levels of influence of age requires a short review of equine-specific dental development. The thick layers of peripheral cementum of the clinical crown devolves underneath the gum line. As continual wear of the equine clinical crown is compensated by a permanent dental eruption, the clinical crown and the peripheral cementum do not age but become replaced continuously. Therefore, destructive lesions of the peripheral cementum do not necessarily worsen over time but can disappear and become replaced by intact, renewed peripheral cementum (2, 6). Jackson et al. (4) also demonstrated that, even in severe cases of peripheral cementum destruction, the destructive process does not extend underneath the junctional epithelium of the gingiva. A resistant junctional epithelium thus guarantees continuous productivity of the subgingival cementation zone.

Due to continuous wear and eruption of the equine tooth, the destructive process on the peripheral cementum is seldomly given time to proceed deeper in the dental structures and reach into the enamel and dentin. The destruction of dentin can possibly lead to an infection of the pulp through oral exposition of dentinal tubules. However, this situation rarely occurs. Thus, destructive disease of the equine peripheral cementum can be considered curable rather than seriously pathological, provided the triggering factors can be eliminated. Conversely, destruction of the equine peripheral cementum differs markedly from human peripheral caries, which is associated with a direct irrecoverable loss of enamel.

In contrast to the continuously renewing peripheral cementum, the infundibular cementum is formed exclusively during the pre-eruptive developmental phase of the tooth. When the tooth erupts through the oral mucosa, the vascular infundibular blood supply is cut off, and all cementum formation ceases (9). These developmental conditions explain a high susceptibility to destructive disease due to a developmental high porosity of the infundibular cementum. In a developmental study (9), it was shown that the mesial infundibulum loses its blood supply earlier than the distal one, which is mirrored by a higher prevalence of incomplete infundibular cemental fillings (23). These findings contribute a plausible explanation for the observed higher prevalence of infundibular destruction in the mesial infundibulum. Furthermore, beyond the background of the distinct developmental conditions, it is understandable that destructive infundibular disease must have a progressively destructive character and therefore feature a marked increasing prevalence with age.

The progression of destructive processes within the infundibular cementum can lead to perforation of the infundibular enamel causing pulpal infection through exposure of neighboring dentinal tubules (24). Such infundibular-borne pulpitis is a well-known equine-specific dental disease (24). Even in cases in which pulpal infection is absent, the advanced destruction of infundibular cementum is regarded as a serious pathological condition as it is a predisposing factor for tooth fractures (25). Although both diseases, peripheral and infundibular destruction, primarily affect dental cementum, and although the same underlining pathological processes are assumed, peripheral and infundibular cemental destruction should be regarded as independent conditions. Accordingly, it is not surprising that there is no statistical correlation between destructive disease of the peripheral cementum and the infundibular cementum. However, 29% of the horses in our study exhibited both types of the destructive disease on the same teeth. A comparable prevalence (32%) of teeth featuring both types of cemental destruction was reported by Gere and Dixon (3).

### 5.5 Topographic predisposition

As already discussed for destructive disorders of the peripheral cementum a spatial predisposition was found in relation to the position of the teeth within the oral cavity, which can be linked to the position of the openings of the salivary glands. For infundibular diseases, however, a predisposition has been demonstrated, but it is related to the Triadan-position of the tooth in the dentition and thus to the duration of tooth development. The most affected teeth, i.e., 09, 10, and 06, are the first to appear in the permanent dentition and have had the least time to develop and mature. Based on studies by Hoppe et al. (26), a period of 11.5 months may be required for tooth 09 to progress from the start of development until it erupts into the oral cavity. For teeth 06 and 10, a corresponding period of approximately 17 months is needed. For all other molars, the period is at least 21 months. Although the formation of new tooth substance continues after eruption of the tooth into the oral cavity, the eruption is a development-limiting event for all processes within the infundibulum, as the vascularization of the infundibulum occurs exclusively from the occlusal side and comes to an abrupt halt as soon as the tooth erupts through the oral mucosa (8, 9). It is hypothesized that a shorter developmental time of the infundibulum is more likely to lead to infundibular hypocementosis and thus to a higher susceptibility to feed impaction and subsequent destructive fermentation processes (23, 27, 28).

The same considerations can also be used as an explanatory hypothesis for the higher prevalence of destructive disease in the mesial infundibulum compared to the distal infundibulum (12, 28). Suske et al. (9) showed that the blood supply to the mesial infundibulum ceases earlier than the blood supply to the distal infundibulum and thus predisposes the mesial infundibulum to higher prevalence of hypocementosis compared to the distal infundibulum.

### 5.6 Diastemata

Our study did not yield any significant correlation between destructive disease of the peripheral cementum and the presence of diastemata. Only a statistical trend was discernible, suggesting that destruction of the peripheral cementum might be associated with diastemata formation. However, studies with larger sample sizes (>500 horses) showed a clear statistical correlation between peripheral cemental defects and diastemata (3, 11).

The prevalence of diastemata was particularly notable in the molar region in the lower and upper jaws, a finding that aligns with the increased prevalence of peripheral changes observed in this area. Therefore, it is likely that destruction of the peripheral cementum also causes destruction of interdental cementum, which, in turn, explains a predisposition for the formation of diastemata between affected teeth. In a recent study, teeth exhibiting moderate to severe peripheral caries lesions were 13 times more likely to develop a diastema than teeth devoid of such lesions (6).

Nevertheless, causative factors other than destruction of the peripheral interdental cementum should be considered in the formation of diastemata. A rise in the prevalence of diastemata with age can be attributed to a decrease in mesio-distal tooth length (29), together with the reduction of the reserve crown and the resultant instability. Furthermore, the occurrence of diastemata is more prevalent in mandibular cheek teeth than in maxillary cheek teeth. This disparity can be attributed, at least in part, to the distinct tooth morphology, with maxillary cheek teeth possessing three roots and mandibular cheek teeth possessing two roots. This disparity in root number gives rise to a heightened degree of instability when comparing upper and lower cheek teeth.

### 5.7 Different grading systems

Regardless of the location of the disease (peripheral or infundibular), previous grading systems (1, 3, 10) focused on the tooth substances, with each tooth substance being considered for destructive lesions individually. Destruction of tooth substances was determined by visually perceptible changes. The disease was graded according to the structures affected, sometimes even including the destroyed pertinent area of a substance. Accordingly, up to five grades and additional subgrades have been defined (1). In view of the complex spatial structure of the horse cheek teeth and the difficulties in visualizing the clinical crown, it is challenging to precisely differentiate between defects in the individual tooth structures. Accordingly, the previous gradation systems are prone to misjudgments. Moreover, with focus only on the individual tooth substances, the clinical significance of a destructive disease is not well reflected. Therefore, an optimized and simplified grading system was developed. To differentiate between substantial defects and superficial discoloration, palpation of suspected areas is recommended to address the clinical relevance of observed changes. Lesions affecting the cementum and enamel were grouped into one grade, while those extending into the dentin, possibly causing pulpal infection, were defined as a separate grade. We emphasize that occlusal inspection and grading of infundibular defects is not sufficient to make a deep diagnosis and justify therapeutic measures. Further examination (e.g., X-ray or computed tomography) should be carried out in addition to determine a well-founded therapeutic approach.

## 6 Conclusion

This study confirms the high prevalence of destructive dental disorders in northern Germany, with peripheral cemental lesions observed in 77.2% and infundibular changes in 61.4% of examined horses. While both conditions affect the dental cementum and show some similarities to human caries in terms of microbial etiology and dietary influences, there is currently no definitive evidence that they constitute true carious disease as defined in human dentistry. The unique anatomy and continuous eruption of equine teeth necessitate species-specific terminology and understanding. Although both disorders begin in the cementum, they differ substantially in their clinical implications. Peripheral cemental defects are typically superficial, often reversible, and have limited clinical impact. In contrast, infundibular lesions can progress into dentin, compromise the pulp, and predispose to tooth fractures, marking them as clinically more significant. These differences warrant the use of separate diagnostic approaches and tailored grading systems that reflect the severity and potential consequences of each lesion type. Furthermore, factors such as age, sex, dental position, and diastemata appear to influence lesion distribution, though some associations did not reach statistical significance. In summary, equine destructive dental diseases require differentiated diagnostic and conceptual frameworks. Continued research is essential to clarify etiological mechanisms, refine classification systems, and improve prevention and management strategies to identify healthy diet for modern horses to the specific conditions of equine oral health.

## Data Availability

The original contributions presented in the study are included in the article/Supplementary material, further inquiries can be directed to the corresponding author.

## Supplementary material

Supplementary Table 1 Patient data (age, breed, sex) and pathological findings, i.e. lesions in the peripheral cementum (LPC), lesions in the infundibula (LI) and occurrence of diastemata within the cheek tooth rows.
