# Supplementary material for: Equine dental destructive disorders: an epidemiological survey in northern Germany
Source: Front Vet Sci. 2026 Feb 17;13:1706621. doi: 10.3389/fvets.2026.1706621 (PMC12954870; doi:10.3389/fvets.2026.1706621)
Supplement: Supplementary Table 1 — Patient data (age, breed, sex) and pathological findings, i.e. lesions in the peripheral cementum (LPC), lesions in the infundibula (LI) and occurrence of diastemata within the cheek tooth rows. [file Table_1.docx]

| Horse number | Age (years) | Breed | Sex | LPC | LI | Diastemata |
| --- | --- | --- | --- | --- | --- | --- |
| 1 | 3 | warmblood | stallion | yes | no | no |
| 2 | 3 | warmblood | stallion | yes | no | no |
| 3 | 3 | warmblood | stallion | no | no | no |
| 4 | 3 | warmblood | stallion | no | no | no |
| 5 | 3 | warmblood | stallion | no | no | no |
| 6 | 3 | warmblood | stallion | no | no | no |
| 7 | 3 | warmblood | stallion | no | no | no |
| 8 | 3 | warmblood | stallion | no | no | no |
| 9 | 3 | warmblood | stallion | no | no | no |
| 10 | 3 | warmblood | stallion | no | no | no |
| 11 | 13 | warmblood | gelding | yes | no | no |
| 12 | 5 | warmblood | gelding | yes | no | yes |
| 13 | 13 | warmblood | gelding | yes | no | yes |
| 14 | 11 | warmblood | mare | yes | no | no |
| 15 | 15 | warmblood | gelding | no | yes | yes |
| 16 | 4 | warmblood | mare | yes | no | yes |
| 17 | 18 | friesian horse | mare | yes | no | no |
| 18 | 17 | friesian horse | mare | yes | no | yes |
| 19 | 8 | quarter horse | gelding | yes | no | no |
| 20 | 5 | warmblood | mare | yes | no | no |
| 21 | 11 | warmblood | gelding | no | no | yes |
| 22 | 8 | warmblood | gelding | no | no | yes |
| 23 | 11 | warmblood | gelding | yes | yes | no |
| 24 | 14 | warmblood | gelding | yes | yes | yes |
| 25 | 8 | warmblood | gelding | no | no | yes |
| 26 | 4 | warmblood | gelding | yes | no | yes |
| 27 | 15 | warmblood | gelding | yes | yes | yes |
| 28 | 10 | warmblood | mare | yes | yes | yes |
| 29 | 7 | quarter horse | gelding | yes | yes | yes |
| 30 | 10 | pony | gelding | no | no | no |
| 31 | 21 | warmblood | mare | yes | yes | yes |
| 32 | 15 | warmblood | gelding | yes | yes | yes |
| 33 | 10 | warmblood | gelding | yes | yes | yes |
| 34 | 17 | warmblood | gelding | yes | no | yes |
| 35 | 14 | warmblood | mare | yes | yes | yes |
| 36 | 19 | warmblood | gelding | yes | yes | yes |
| 37 | 6 | warmblood | gelding | yes | yes | yes |
| 38 | 7 | warmblood | gelding | yes | yes | yes |
| 39 | 9 | quarter horse | mare | yes | yes | no |
| 40 | 15 | warmblood | mare | yes | yes | yes |
| 41 | 8 | warmblood | mare | yes | yes | yes |
| 42 | 8 | friesian horse | gelding | yes | no | no |
| 43 | 12 | warmblood | stallion | yes | yes | yes |
| 44 | 4 | warmblood | mare | yes | no | yes |
| 45 | 9 | warmblood | gelding | no | no | no |
| 46 | 6 | warmblood | gelding | yes | no | no |
| 47 | 19 | warmblood | stallion | no | yes | no |
| 48 | 6 | warmblood | gelding | yes | no | no |
| 49 | 7 | warmblood | stallion | no | yes | no |
| 50 | 3 | warmblood | gelding | yes | no | no |
| 51 | 14 | warmblood | gelding | yes | yes | yes |
| 52 | 6 | warmblood | mare | no | no | no |
| 53 | 7 | warmblood | gelding | no | no | no |
| 54 | 6 | warmblood | gelding | no | yes | no |
| 55 | 5 | warmblood | mare | no | yes | no |
| 56 | 13 | warmblood | gelding | yes | yes | yes |
| 57 | 7 | warmblood | gelding | yes | yes | no |
| 58 | 3 | warmblood | mare | yes | no | yes |
| 59 | 9 | warmblood | gelding | yes | yes | yes |
| 60 | 3 | warmblood | stallion | yes | no | no |
| 61 | 6 | warmblood | mare | yes | no | yes |
| 62 | 19 | warmblood | mare | yes | no | yes |
| 63 | 3 | warmblood | mare | yes | no | yes |
| 64 | 10 | warmblood | mare | no | no | no |
| 65 | 17 | friesian horse | gelding | yes | yes | no |
| 66 | 6 | warmblood | mare | no | yes | no |
| 67 | 8 | spanish horse | gelding | yes | no | yes |
| 68 | 15 | warmblood | gelding | yes | no | no |
| 69 | 9 | friesian horse | stallion | yes | yes | yes |
| 70 | 7 | warmblood | mare | yes | yes | yes |
| 71 | 8 | friesian horse | mare | yes | yes | yes |
| 72 | 5 | paint horse | gelding | yes | yes | no |
| 73 | 9 | warmblood | mare | yes | yes | no |
| 74 | 3 | warmblood | mare | yes | no | no |
| 75 | 10 | warmblood | gelding | yes | yes | no |
| 76 | 13 | pony | gelding | yes | yes | no |
| 77 | 5 | warmblood | mare | yes | yes | no |
| 78 | 11 | warmblood | mare | yes | yes | yes |
| 79 | 10 | warmblood | gelding | yes | yes | yes |
| 80 | 13 | pony | gelding | yes | yes | no |
| 81 | 24 | pony | gelding | no | yes | yes |
| 82 | 12 | warmblood | mare | yes | yes | no |
| 83 | 3 | warmblood | gelding | yes | no | no |
| 84 | 4 | warmblood | mare | yes | yes | yes |
| 85 | 10 | spanish horse | gelding | yes | yes | yes |
| 86 | 4 | warmblood | mare | yes | yes | yes |
| 87 | 14 | friesian horse | gelding | yes | yes | yes |
| 88 | 13 | warmblood | mare | yes | yes | yes |
| 89 | 5 | warmblood | gelding | no | yes | yes |
| 90 | 6 | warmblood | mare | yes | yes | yes |
| 91 | 19 | warmblood | mare | yes | yes | yes |
| 92 | 19 | pony | mare | yes | yes | yes |
| 93 | 18 | warmblood | gelding | yes | yes | yes |
| 94 | 17 | warmblood | mare | yes | yes | yes |
| 95 | 23 | quarter horse | mare | yes | yes | yes |
| 96 | 24 | pony | mare | yes | yes | yes |
| 97 | 22 | warmblood | gelding | yes | yes | no |
| 98 | 15 | warmblood | gelding | yes | yes | no |
| 99 | 18 | warmblood | gelding | yes | yes | no |
| 100 | 18 | warmblood | gelding | yes | yes | no |
| 101 | 22 | warmblood | gelding | no | yes | no |
| 102 | 21 | pony | gelding | yes | yes | yes |
| 103 | 24 | pony | mare | yes | yes | yes |
| 104 | 17 | paint horse | mare | yes | yes | yes |
| 105 | 26 | pony | gelding | yes | yes | yes |
| 106 | 18 | warmblood | mare | no | no | yes |
| 107 | 18 | warmblood | mare | yes | yes | yes |
| 108 | 16 | warmblood | mare | yes | yes | yes |
| 109 | 16 | warmblood | mare | yes | yes | yes |
| 110 | 15 | warmblood | gelding | yes | yes | yes |
| 111 | 22 | warmblood | gelding | yes | yes | yes |
| 112 | 15 | warmblood | gelding | yes | yes | yes |
| 113 | 18 | warmblood | gelding | yes | yes | no |
| 114 | 16 | pony | mare | yes | yes | no |

Supplementary Table 1
